# Physician-Modified Endografts in the Treatment of Aortic Arch Disease: The Need for Unity and Standardization

**DOI:** 10.1177/15266028251388737

**Published:** 2025-10-29

**Authors:** Emiel W. M. Huistra, Ludovic Canaud, Clark J. Zeebregts

**Affiliations:** 1 Department of Surgery, Division of Vascular Surgery, University Medical Center Groningen, University of Groningen, Groningen, The Netherlands; 2 Department of Thoracic and Vascular Surgery, Arnaud de Villeneuve Hospital, Montpellier, France

**Keywords:** physician-modified endovascular graft, TEVAR, thoracic aorta, aortic arch, endovascular arch repair

The term Physician-Modified Endovascular Graft (PMEG), used to describe the process of back-table modification of an endograft with fenestrations or branches, was first coined by Starnes in 2012.^1^ In retrospect, the first fenestrated endovascular aortic repair, performed in 1996, was in fact physician-modified and be retrospectively considered as a PMEG.^2^ Now, nearly 3 decades later, PMEG remains a valuable tool in the armamentarium of vascular surgeons managing complex aortic pathologies, despite the emergence of custom-made (CMD) and off-the-shelf fenestrated and branched devices.^3^

Technological improvements in PMEG techniques have enabled reinforcement of fenestrations, addition of mini-cuffs, and the incorporation of preloaded guidewires.^4^ These advancements have expanded the applicability of PMEGs, extending their use even into complex aortic arch repair. This raises the question of what the current and future roles of PMEG in arch repair comprises, particularly in an era with an increasing availability of custom-made and off-the-shelf endovascular devices.

## Is There a Role for PMEG in the Aortic Arch?

Open surgical repair remains the gold standard for aortic arch disease in patients fit for major surgery.^5,6^ However, those deemed unfit may benefit from an endovascular alternative. Standard thoracic endovascular aortic repair (TEVAR) with coverage of the left subclavian artery can be performed to extend the sealing zone 2.^7^ This approach is not applicable to zones 0 and 1. Moreover, coverage of the left subclavian artery is associated with increased neurologic complications, therefore, maintaining left subclavian artery perfusion is generally preferred.^8^

Hybrid procedures, such as carotid-carotid with carotid-left subclavian bypass, offer another solution. However, this approach is still more invasive than fully endovascular alternatives, carries risks such as phrenic nerve palsy, and would require a sternotomy for zone 0 repairs.^9^

Fenestrated and branched CMD offer an alternative.^10^ These devices allow for a fully endovascular approach while maintaining antegrade perfusion. The primary limitation of CMDs is their manufacturing lead time, prohibiting their use in urgent situations. In such cases, PMEG, in situ fenestration, or parallel grafting may be the only viable alternatives.

While acceptable initial technical success with parallel endografts and in situ fenestration can be achieved, there are considerable downsides to these techniques. Parallel grafting has a high risk of type 1 “gutter” endoleak, especially when multiple parallel grafts are used.^11^ In situ fenestrations lack reinforcement, which may compromise durability and lead to type 3 endoleak, particularly under the forces exerted by motion in the aortic arch, forces that may, at least theoretically, favor the use of branches over fenestrations.^12–14^ In contrast, PMEG best replicates CMD principles. Despite the potential benefits, the adoption of PMEG in arch repair has been limited due to the technical challenge, and lack of high-quality evidence.^15^

Recent studies have reinforced the potential of PMEG in aortic arch repair. The effectiveness of PMEG for zone 0–2 repairs for a wide range of aortic pathologies has been demonstrated in small single center cohorts.^16–19^ Bacri et al^20^ reported 100% technical success in 74 patients with zone 0 PMEG for degenerative aneurysms, post dissection aneurysms and acute type B dissections. The study showed a 5% stroke rate, 1% early mortality, and 9% early reintervention. During a 40.7-month mean follow-up, 1 type 1 endoleak and 4 reinterventions were reported.^20,21^ Wen et al^22^ confirmed these results in 54 patients across 3 centers. Technical success was 98%, stroke occurred in 7%, early mortality was 4%, and during 12 months median follow-up, 7 patients had type 1/3 endoleak and 10 patients required reintervention.^22^ These outcomes are similar to inner-branched CMDs for zone 0 repairs, where 100% technical success, 5% stroke, and 5% early mortality were reported in 39 patients.^23^

The largest series on PMEG in the arch, by Li et al,^24^ includes 513 patients from 7 centers. Technical success was 98.6%, with 2.5% early mortality and 2.3% stroke at 30 days. Results were durable after a median 27 months follow-up, with 2.6% requiring reintervention. However, the study’s generalizability is limited by the inclusion of a broad range of pathologies and proximal extent of disease (including zone 0–4). It should be noted that the stroke rate is exceptionally low compared with other reports and may not reflect contemporary real-world outcomes.^10^

There is the recent emergence of branched off-the-shelf endografts to consider as an alternative, with potential broad application due to considerable overlap between patient anatomies in the arch.^25^ However, this “one-size-fits-most” solution leaves a subset of patients with unsuitable anatomy with less desirable options, like parallel grafting or in situ fenestration, and physicians that are less familiar with these alternatives as a consequence of using off-the-shelf branched option in most patients. In addition, there are some recent concerns about branch occlusion during follow-up. During a median follow-up of 20.0 months, Lemmens et al^26^ reported a late stroke rate of 23% in patients treated with an arch branched device, underscoring that the risk of stroke does not end after the procedure. Performing PMEG only in a very select acute group of patients will generally not allow for sufficient number of cases to maintain proficiency of this technically demanding technique. Let alone perform the modifications in a timely and consistent manner under stressful conditions. By maintaining a robust PMEG program, consistent modification times under 25 minutes have been reported.^27^ Rather than pushing the envelope with off-the-shelf devices, performing PMEG for patients not amenable to a CMD due to waiting time or financial constraints might overall benefit the largest patient population.

The advantages of PMEG are clear: a patient-specific treatment for different anatomies, that can be achieved timely, significantly reducing the costs compared with CMD, and favorable outcomes in preliminary research compared with in situ fenestration and parallel endograft techniques. It seems that PMEG is here to stay. However, difficulties remain.

## Current Issues Regarding PMEG

Despite growing interest and promising short-term to mid-term outcomes, several challenges currently limit the widespread adoption of PMEG in aortic arch repair.

First, PMEG is an umbrella term encompassing a plethora of modification techniques. Variations exist in the materials used to reinforce fenestrations (wire tips, snare loops, coils), the presence or absence of diameter reducing ties (and the technique to add them), the use of preloaded guidewires, and the configuration of fenestration, ranging from multiple small fenestrations for each of the major arch vessels to a large fenestrations for 2 major branches.^20,24,28–32^ In addition, there is variation in stenting of 1 or all of the target vessels, and which endograft platform is used. This lack of a standardized technique makes it difficult to generalize results across centers and complicates comparative outcome analysis.

Second, while recent studies have shown durable results up to 3 years postprocedure, there is no guarantee for long-term success.^33^ Elucidating long-term outcomes is of particular importance due to questions regarding the structural integrity of endografts after modifications made outside the manufacturer’s intended use.

Third, modification of off-the-shelf devices is off-label, placing full responsibility on the surgeon. Ideally, PMEG should be used after approval through an institutional review board or within an investigational device exemption trial.^34,35^ The legal and ethical implications of using off-label modifications require careful consideration and informed consent by the patient.

Fourth, the current literature is limited to single center retrospective studies. A larger multicenter retrospective study on this topic was hampered by the heterogeneous population, while each distinct pathology and extent of disease (zone 0–4) will have different technical requirements and outcomes. These positive results all come from high volume centers. Overcoming the learning curve seems crucial in arch repairs to reduce the risk of stroke.^24^ Therefore, only limited recommendations can be drawn from the current evidence, prohibiting the uptake of PMEG in treatment guidelines, and requiring careful consideration in light of legal issues.

## Where to Go From Here?

Unity is the way forward. Standardization of techniques is the key to establish PMEG as a validated treatment strategy for complex aortic arch pathologies, with reproducible outcomes, cost-benefit analysis, and valid comparisons with alternative treatment strategies. Important questions remain, including which techniques used to modify devices yield the most reliable results, what platform to use, how many procedures are required to maintain proficiency, and what are the long-term outcomes of PMEG. Vascular specialists should work toward a consensus on the indications, planning, techniques, and platforms used in PMEG. Establishing prospective registries focused on standardized techniques will be key in answering these questions.

Progress is clearly being made, for example, the reporting of PMEG protocols, and the creation of a standardized nitinol ring that mimics the design of a CMD.^31^ Collaborative initiatives regarding the treatment of complex abdominal aortic disease provide an example of what is possible.^36^ Tsilimparis et al^37^ reported positive results for 1274 patients across 19 centers which were retrospectively analyzed, and Piazza et al^38^ collected prospective multicenter data of a standardized approach to PMEG for complex AAA, demonstrating excellent technical implantation safety and efficacy. The authors highlight that their efforts can serve as a benchmark for future comparison with other PMEG, off-the-shelf, or custom endografts. Involvement of industry in standardizing PMEG, through dedicated platforms and standardized tools, will further enhance the consistency and reproducibility of PMEG.

Although CMD remains the gold standard for endovascular repair of the arch, they may be unavailable due to time constraints, logistical barriers, and costs. In such cases, PMEG offers a viable alternative. As long as PMEG remains a catch-all term encompassing a broad spectrum of techniques and modifications, its full potential will be difficult to define or compare with regulated alternatives. Unity is the way forward. Standardization is the key to evolve PMEG into an evidence-based, guideline-supported option, and allow for valid comparison between different treatment strategies, or even on-label use of strategies involving modifications of endografts.

## References

[1] Starnes BW. Physician-modified endovascular grafts for the treatment of elective, symptomatic, or ruptured juxtarenal aortic aneurysms. J Vasc Surg. 2012;56:601–607. 10.1016/j.jvs.2012.02.011 22554425

[2] Park JH Chung JW Choo IW , et al. Fenestrated stent-grafts for preserving visceral arterial branches in the treatment of abdominal aortic aneurysms: preliminary experience. J Vasc Interv Radiol. 1996;7(6):819–823. 10.1016/s1051-0443(96)70854-0 8951748

[3] Canonge J Jayet J Heim F , et al. Comprehensive review of physician modified aortic stent grafts: technical and clinical outcomes. Eur J Vasc Endovasc Surg. 2021;61:560–569. 10.1016/j.ejvs.2021.01.019 33589325

[4] Oderich GS. Commentary: physician-modified vs off-the-shelf fenestrated and branched endografts: is this a fair comparison? J Endovasc Ther. 2016;23(1):110–114. 10.1177/1526602815613520 26763258

[5] Patel HJ Nguyen C Diener AC , et al. Open arch reconstruction in the endovascular era: analysis of 721 patients over 17 years. J Thorac Cardiovasc Surg. 2011;141(6):1417–1423. 10.1016/j.jtcvs.2011.02.020 21459389

[6] Czerny M Schmidli J Adler S , et al. Editor’s choice—current options and recommendations for the treatment of thoracic aortic pathologies involving the aortic arch: an expert consensus document of the European Association for Cardio-Thoracic Surgery (EACTS) & the European Society for Vascular Surgery (ESVS). Eur J Vasc Endovasc Surg. 2019;57(2):165–198. 10.1016/j.ejvs.2018.09.016 30318395

[7] Rizvi AZ Murad MH Fairman RM , et al. The effect of left subclavian artery coverage on morbidity and mortality in patients undergoing endovascular thoracic aortic interventions: a systematic review and meta-analysis. J Vasc Surg. 2009;50(5):1159–1169. 10.1016/j.jvs.2009.09.002 19878792

[8] Hajibandeh S Hajibandeh S Antoniou SA , et al. Meta-analysis of left subclavian artery coverage with and without revascularization in thoracic endovascular aortic repair. J Endovasc Ther. 2016;23:634–641. 10.1177/1526602816651417 27225214

[9] Voigt SL Bishawi M Ranney D , et al. Outcomes of carotid-subclavian bypass performed in the setting of thoracic endovascular aortic repair. J Vasc Surg. 2019;69(3):701–709. 10.1016/j.jvs.2018.07.022 30528402

[10] Basha AM Moore RD Rommens KL , et al. A systematic review of total endovascular aortic arch repair: a promising technology. Can J Cardiol. 2023;39(1):49–56. 10.1016/j.cjca.2022.11.003 36395997

[11] Mangialardi N Serrao E Kasemi H , et al. Chimney technique for aortic arch pathologies: an 11-year single-center experience. J Endovasc Ther. 2014;21(2):312–323. 10.1583/13-4526MR.1 24754293

[12] Ahanchi SS Almaroof B Stout CL , et al. In situ laser fenestration for revascularization of the left subclavian artery during emergent thoracic endovascular aortic repair. J Endovasc Ther. 2012;19:226–230. 10.1583/11-3770MR.1 22545888

[13] Redlinger RE Jr Ahanchi SS Panneton JM. In situ laser fenestration during emergent thoracic endovascular aortic repair is an effective method for left subclavian artery revascularization. J Vasc Surg. 2013;58(5):1171–1177. 10.1016/j.jvs.2013.04.045 23746832

[14] Kopp R Katada Y Kondo S , et al. Multicenter analysis of endovascular aortic arch in situ stent-graft fenestrations for aortic arch pathologies. Ann Vasc Surg. 2019;59:36–47. 10.1016/j.avsg.2019.02.005 31009715

[15] Atkins MD Lumsden AB. Parallel grafts and physician modified endografts for endovascular repair of the aortic arch. Ann Cardiothorac Surg. 2022;11(1):16–25. 10.21037/acs-2021-taes-171 PMC8807419 35211381

[16] Bacri C Hireche K Alric P , et al. Total aortic arch repair with double-fenestrated physician-modified endograft under local anesthesia and sedation: a prospective study [published online ahead of print December 7, 2024]. J Endovasc Ther. doi:10.1177/15266028241302746. 39644174

[17] Bacri C Hireche K Alric P , et al. Ascending aorta anastomotic aneurysm exclusion with double-fenestrated PMEG after open repair. Ann Vasc Surg. 2024;109:396–406. 10.1016/j.avsg.2024.07.093 39096954

[18] Lounes Y Belarbi A Hireche K , et al. Physician-modified stent graft for blunt thoracic aortic injuries: do the benefits worth the trouble? Ann Vasc Surg. 2023;90:100–108. 10.1016/j.avsg.2022.09.054 36270548

[19] Moqaddam M Bacri C Hireche K , et al. Short-term results of fenestrated physician-modified endografts for type1a endoleak after conventional thoracic endovascular aortic repair. JTCVS Tech. 2024;25:8–18. 10.1016/j.xjtc.2024.03.003 PMC11184594 38899088

[20] Bacri C Hireche K Alric P , et al. Total aortic arch repair with double-fenestrated physician-modified endografts, at least 3-year follow-up. J Vasc Surg. 2024;80(2):344–354. 10.1016/j.jvs.2024.03.029 38552884

[21] Canaud L Chassin-Trubert L Abouliatim I , et al. Total arch thoracic endovascular aortic repair using double fenestrated physician-modified stent-grafts: 100 patients. J Endovasc Ther. 2024;31(1):89–97. 10.1177/15266028221116747 35927926

[22] Wen Q Zhang Y Wei J , et al. Physician modified fenestrated endografts for endovascular aortic arch repair in zone 0. Eur J Vasc Endovasc Surg. 2024;68(2):190–199. 10.1016/j.ejvs.2024.04.008 38641315

[23] Tenorio ER Oderich GS Kölbel T , et al. Multicenter global early feasibility study to evaluate total endovascular arch repair using three-vessel inner branch stent-grafts for aneurysms and dissections. J Vasc Surg. 2021;74(4):1055–1065.e4. 10.1016/j.jvs.2021.03.029 33865950

[24] Li X Li W Dai X , et al. Thoracic endovascular repair for aortic arch pathologies with surgeon modified fenestrated stent grafts: a multicentre retrospective study. Eur J Vasc Endovasc Surg. 2021;62(5):758–766. 10.1016/j.ejvs.2021.07.017 34629276

[25] Bosse C Kölbel T Mougin J , et al. Off-the-shelf multibranched endograft for total endovascular repair of the aortic arch. J Vasc Surg. 2020;72(3):805–811. 10.1016/j.jvs.2019.11.046 32151480

[26] Lemmens CC Konings TJAJ Dean A , et al. Branch thrombus after endovascular treatment with arch branched devices for aortic arch pathologies. Eur J Vasc Endovasc Surg. 2025;69(1):38–45. 10.1016/j.ejvs.2024.07.023 39053643

[27] Chastant R Belarbi A Ozdemir BA , et al. Homemade fenestrated physician-modified stent grafts for arch aortic degenerative aneurysms. J Vasc Surg. 2022;76(5):1133–1140.e2. 10.1016/j.jvs.2022.04.041 35697312

[28] Zhang Y Shen J Yang P , et al. Physician-modified endograft with triple inner branches for extensive aortic arch aneurysm. J Endovasc Ther. 2022;29(4):623–626. 10.1177/15266028211059439 34839726

[29] Papazoglou DD Karaolanis GI Kotelis D , et al. Physician-modified endografts with the TREO stent graft system [published online ahead of print September 26, 2024]. J Endovasc Ther. doi:10.1177/15266028241282643. 39323299

[30] Huistra EWM Saers S Fokkema TM , et al. Technical approach to rescuing a previous physician-modified endovascular graft with a new physician-modified endovascular graft [published online ahead of print March 14, 2025]. J Endovasc Ther. doi:10.1177/15266028251326844. 40084849

[31] Csobay-Novák C Juhos B Szentiványi A , et al. A standardized physician-modified endograft workflow utilizing the punch card technique and the Hungaroring reinforcement to treat complex abdominal aortic aneurysms. J Vasc Surg Cases Innov Tech. 2024;11:101649. 10.1016/j.jvscit.2024.101649 PMC11617762 39649730

[32] Huistra EWM Alrawi W Tielliu IFJ , et al. Preloaded physician-modified thoracic endovascular aortic repair to achieve left subclavian artery fenestration alignment in zone 2 [published online ahead of print June 18, 2025]. J Endovasc Ther. doi:10.1177/15266028251344882. 40530603

[33] Karaolanis GI Makaloski V Jungi S , et al. Endovascular repair of aortic arch zones 0 to 2 using physician-modified endografts: a systematic review and meta-analysis [published online ahead of print November 8, 2024]. J Endovasc Ther. doi:10.1177/15266028241292485. 39513511

[34] Starnes BW. A surgeon’s perspective regarding the regulatory, compliance, and legal issues involved with physician-modified devices. J Vasc Surg. 2013;57(3):829–831. 10.1016/j.jvs.2012.11.043 23446124

[35] Ricci G Gibelli F Sirignano A , et al. Physician-modified endografts for repair of complex abdominal aortic aneurysms: clinical perspectives and medico-legal profiles. J Pers Med. 2024;14:759. 10.3390/jpm14070759 PMC11278265 39064014

[36] Robaldo A Ruffino MA Garbero E , et al. Indications, planning, and technical aspects in physician-modified endografts based on a cross-sectional global survey. J Vasc Surg. 2025;82(3):760–769.e9. 10.1016/j.jvs.2025.05.019 40404023

[37] Tsilimparis N Gouveia E Melo R , et al. Multicenter study on physician-modified endografts for thoracoabdominal and complex abdominal aortic aneurysm repair. Circulation. 2024;150:1327–1342. 10.1161/CIRCULATIONAHA.123.068587 38989565

[38] Piazza M Spertino A Colacchio EC , et al. Early outcomes from the Multicentre Standardised PHysician-Modified 4-Fenestrated Endograft REgistry (S.PH.E.RE.) [published online ahead of print June 3, 2025]. Eur J Vasc Endovasc Surg. doi:10.1016/j.ejvs.2025.05.057. 40472964

